# A Nomogram for Predicting Cardiovascular Diseases in Chronic Obstructive Pulmonary Disease Patients

**DOI:** 10.1155/2022/6394290

**Published:** 2022-10-18

**Authors:** Shuang Qu, Jing Zhu

**Affiliations:** Department of Respiration, Fu Xing Hospital, Capital Medical University, Beijing, China

## Abstract

Cardiovascular diseases (CVDs) are the most common comorbidities in the chronic obstructive pulmonary disease (COPD), which increase the risk of hospitalization, length of stay, and death in COPD patients. This study aimed to identify the predictors for CVDs in COPD patients and construct a prediction model based on these predictors. In total, 1022 COPD patients in National Health and Nutrition Examination Surveys (NHANES) were involved in the cross-sectional study. All subjects were randomly divided into the training set (*n* = 709) and testing set (*n* = 313). The differences before and after the manipulation of the missing data were compared via sensitivity analysis. Univariate and multivariable analyses were employed to screen the predictors of CVDs in COPD patients. The performance of the prediction model was evaluated via the area under the curve (AUC), accuracy, sensitivity, specificity, negative predictive value (NPV), positive predictive value (PPV), and calibration. Subgroup analysis was performed in patients using different COPD diagnosis methods and patients smoking or not smoking in the testing set. We found that male, older age, a smoking history, overweight, a history of blood transfusion, a history of heart disease in close relatives, higher levels of white blood cell (WBC), and monocyte (MONO) were associated with the increased risk of CVDs in COPD patients. Higher levels of platelets (PLT) and lymphocyte (LYM) were associated with reduced risk of CVDs in COPD patients. A prediction model for the risk of CVDs in COPD patients was established based on predictors including gender, age, a smoking history, BMI, a history of blood transfusion, a history of heart disease in close relatives, WBC, MONO, PLT, and LYM. The AUC value of the prediction model was 0.75 (95% CI: 0.71–0.79) in the training set and 0.79 (95%CI: 0.73–0.85) in the testing set. The prediction model established showed good predictive performance in predicting CVDs in COPD patients.

## 1. Introduction

As a complex respiratory disorder, chronic obstructive pulmonary disease (COPD) is characterized by persistent airflow limitation associated with an abnormal inflammatory due to exposure to noxious particles and gases [1, 2]. The prevalence of COPD is 11%–26%, and the worrisome trend is expected to continue over the next 25 years [3]. Alarmingly, over 6 million deaths were estimated to result from COPD annually all over the world, and by 2030, COPD will become the third major reason of death all through the world [4]. This prediction has already been fulfilled and COPD has been reported to have caused 3.23 million deaths in 2019 [5]. The prevalence of COPD has caused a large burden to the society with an estimated cost of US$50 trillion per year [6]. COPD is expected to become the main economic burden of human chronic diseases in the future with the increase in air pollution and the speed of aging worldwide [7]. To display special concern on COPD was essential for the society and patients.

Although COPD primarily affects the lungs, patients also suffered from concurrent comorbidities such as cardiovascular diseases (CVDs), lung cancers, and metabolic diseases [8]. CVDs are the most common comorbidities in COPD, which increase the risk of hospitalization, length of stay, and death in COPD patients [9]. Previously, studies have reported that the prevalence of CVDs in COPD patients was approximately 10%–38% [10], and CVDs caused about 20%–50% of mortality in COPD patients [11]. To prevent the occurrence of CVD in COPD patients was of vital significance for improving the prognosis of those patients.

Accumulating research findings emerged over the past years, and the risk factors for CVDs in COPD patients were identified in various studies. Increased serum levels of inflammation and oxidative stress associated factors such as vascular cell adhesion molecule-1 [12] and human epididymis protein 4 [13] were reported to be correlated with the increased risk of CVDs in COPD patients. Chronic bronchial infection was also identified to increase the incidence of CVDs in COPD patients [14]. Machine learning enables systems to automatically learn and build the analytical model from their experience, and various prediction models were built for identifying those with the risk of some diseases, or for clinical use [15, 16]. The prediction models provided valuable tools for helping and guiding the treatments and care for clinicians and nurses. Previously, a prediction model for predicting the risk of CVDs in COPD patients was also established based on monocyte (MONO) level/HDL cholesterol ratio with an area under the receiver operating characteristic curve (AUC) of 0.73 [17]. This model only focused on the MONO level/HDL cholesterol ratio in those patients, which lacked important demographic and clinical variables associated with inflammation in COPD [18]. In this study, we collected the data of 1022 COPD patients from the National Health and Nutrition Examination Surveys (NHANES) between 2007 and 2018. The purpose of our study was to explore the predictors for CVDs in COPD patients and construct a prediction model based on these predictors. A nomogram for predicting CVDs in COPD patients was also plotted to quickly identify the possibility of CVDs in COPD patients.

## 2. Methods

### 2.1. Study Population

**Figure 1:**
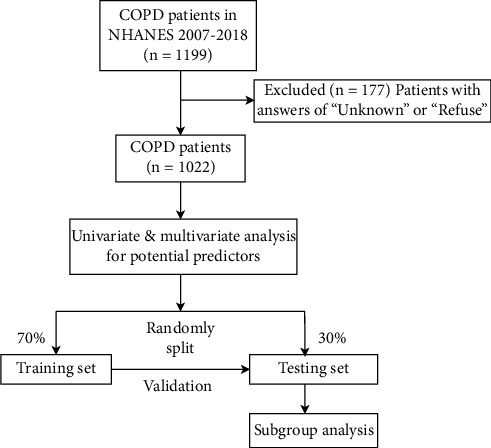
The screening process of subjects in this study.

The current cross-sectional study collected the data of 1199 COPD patients in the NHANES database from 2007 to 2018. The NHANES is an ongoing program performed by the National Center for Health Statistics (NCHS) of the Centers for Disease Control (CDC) to evaluate the health and nutritional status in the civilian noninstitutionalized populations of the United States [19]. Therefore, informed consent from the participants was waived. Every year, about 5000 nationally representative individuals are sampled through multistage, stratified, clustered sampling method [19]. In our study, patients with answers “unknown” or “refuse” were excluded and finally 1022 patients were included. All participants were divided into the training set (*n* = 709) and the testing set (*n* = 313). The screening process of subjects is displayed in Figure 1.

### 2.2. Main Variables and Outcome Variables

The main variables analyzed included age (years), gender, race (non-Hispanic White, non-Hispanic Black, or other race), education level (under 12th grade, high school grad/general equivalent diploma or equivalent, or some college or above), marital status (married or other), annual family income (<20000$ or ≥20000$), smoke or not, overweight or not, a history of blood transfusion, a history of heart disease in close relatives, white blood cell (WBC; 10^9^/L), MONO (10^9^/L), neutrophil (NEUT; 10^9^/L), platelet (PLT; 10^9^/L), lymphocyte (LYM; 10^9^/L), NEUT/LYM ratio (NLR), and PLT/LYM ratio (PLR).

The outcome variable was COPD patients with CVDs. The COPD was defined according to the question MCQ160O (Has a doctor or other health professional ever told that you had COPD?) in the MCQ series with an answer of “Yes” or verified by spirometry with postbronchodilator forced expiratory volume in 1 second (FEV1)/forced vital capacity (FVC) ratio (FEV1/FVC) ≤0.7 [20]. CVDs were defined as patients with at least one myocardial infarction, congestive heart failure, angina pectoris, or a history of coronary heart disease.

### 2.3. Statistical Analysis

The Kolmogorov–Smirnov test was used to assess the normality of the measurement data. Continuous variables of normal distribution were represented by mean standard deviation (Mean ± SD), and comparison between groups was performed by *t*-test. The measurement data of nonnormal distribution were exhibited as *M* (*Q*_1_, *Q*_3_), and differences between groups were compared via Wilcoxon rank sum test. The enumeration data were described as *n* (%), and chi-square (*χ*^2^) or Fisher's exact probability method were applied for comparisons between groups [21]. The differences before and after the manipulation of the missing data were compared in the sensitivity analysis. All subjects were classified into the training set and the testing set with a ratio of 7 : 3. Univariate and multivariable analyses were employed to screen the predictors of CVDs in COPD patients. The prediction model was constructed and the nomograms were plotted. The evaluation of the prediction model performance was performed via the area under the curve (AUC), accuracy, sensitivity, specificity, negative predictive value (NPV), positive predictive value (PPV), and calibration. The receiver operator characteristic (ROC) curves were drawn, and subgroup analysis was performed in patients using different COPD diagnosis methods and patients smoking or not smoking in the testing set. Statistical analysis was conducted via SAS 9.4 software and R4.0.2 software was used to construct the model. *P* < 0.05was considered as a statistical difference.

### 2.4. The Proposed Architecture of Our Study

In this study, the data of 1022 COPD patients from the NHANES between 2007 and 2018 were collected. The purpose of our study was to explore the predictors for CVDs in COPD patients and construct a prediction model based on these predictors. All subjects were classified into the training set and the testing set with a ratio of 7 : 3. Univariate and multivariable analyses were employed to screen the predictors of CVDs in COPD patients. The prediction model was constructed, and a nomogram for predicting CVDs in COPD patients was also plotted to quickly identify the possibility of CVDs in COPD patients. Our prediction model had good predictive performance and the nomogram made it easy for the clinicians to quickly estimate the possibility of CVDs in a COPD patient and provide timely interventions to prevent the occurrence of CVDs in patients with high risk of CVDs.

## 3. Results

### 3.1. The Manipulation of Missing Data

Variables with a missing value were filled with the median. Sensitivity analysis was performed to compare the data before and after filling. The results revealed that no statistical difference was observed in the data before and after filling the median (Supplementary Table 1).

### 3.2. Comparison of the Characteristics between COPD Patients with CVDs and without CVDs

As depicted in Table 1, the mean age (60.34 years vs. 66.33 years, *t* = −7.38, *P* < 0.001), mean level of WBC (7.62 10^9^/L vs. 8.08 10^9^/L, *t* = -2.89, *P* = 0.004), and MONO (8.16 10^9^/L vs. 8.75 10^9^/L, *t* = −3.30, *P* < 0.001) in patients without CVDs were lower than patients with CVDs. The mean PLT level in patients without CVDs were lower than patients with CVDs (244.59 10^9^/L vs. 228.70 10^9^/L, *t* = 3.43, *P* < 0.001). The average level of NEUT (4.40 vs. 4.80, *Z* = 3.289, *P*=0.001) and NLR (2.19 vs. 2.55, *Z* = 4.577, *P* < 0.001) in patients without CVDs were lower than patients with CVDs. The percentages of patients in terms of education level (*Z* = −2.139, *P*=0.032), annual family income (*χ*^2^ = 7.599, *P*=0.006), smoking (*χ*^2^ = 8.817, *P*=0.003), overweight (*χ*^2^ = 25.203, *P* < 0.001), a history of blood transfusion (*χ*^2^ = 48.405, *P* < 0.001), and a history of heart disease in close relatives (*χ*^2^ = 31.967, *P* < 0.001) were statistically different between patients with CVDs and without CVDs.

### 3.3. Predictors of CVDs in COPD Patients

Variables with statistical significance in the baseline data of COPD patients with or without CVDs were included in the multivariate logistic regression analysis. The results showed that males were associated with 1.060-fold higher risk of CVDs than females (odd ratios (OR) = 1.060, 95% confidence interval (CI): 0.752–1.494). Increased age in COPD patients was associated with a higher risk of CVDs (OR = 1.040, 95% CI: 1.025–1.055). A smoking history in COPD patients increased the risk of CVDs by 0.737 times (OR = 1.737, 95% CI: 1.118–2.697). The risk of CVDs was increased by 0.987 times in overweight patients (OR = 1.987, 95% CI: 1.449–2.725). Patients with a history of blood transfusion were associated with 2.437-fold higher risk of CVDs (OR = 2.437, 95% CI: 1.734–3.425). A history of heart disease in close relatives increased the risk of CVDs by 1.758 times in COPD patients (OR = 2.758, 95% CI: 1.919–3.962). Higher levels of WBC (OR = 1.227, 95% CI: 1.125–1.338) and MONO (OR = 1.085, 95% CI: 1.016–1.159) were associated with a higher risk of CVDs in COPD patients. Higher levels of PLT (OR = 0.996, 95% CI: 0.993–0.998) and LYM (OR = 0.723, 95% CI: 0.568–0.920) were associated with a decreased risk of CVDs in COPD patients (Table 2).

### 3.4. The Equilibrium Test of the Training Set and Testing Set

The participants were randomly divided into the training set and the testing set (7 : 3). The results of equilibrium analysis revealed that there was no statistical significance in the differences of variables between the training set and the testing set (all *P* > 0.05) (Table 3), which indicated that the data in the training set and the testing set were almost equilibrated.

### 3.5. Construction of the Logistic Prediction Model and Validation of the Predicative Value via the Testing Set

Predictors with statistical difference in the multivariable regression analysis were involved in the logistic prediction model. The results delineated those males had a 1.231-fold higher risk of CVDs than females (OR = 1.231, 95% CI: 0.817–1.856). COPD patients with older ages were correlated with a 1.037-fold increase of the risk of CVDs (OR = 1.037, 95% CI: 1.019–1.054). COPD patients with a smoking history were associated with a 1.497-fold increase of the risk of CVDs (OR = 1.497, 95% CI: 0.891–2.513). Overweight was linked with a 1.575-fold increase of the risk of CVDs in COPD patients (OR = 1.575, 95% CI: 1.080–2.298). Patients with a history of blood transfusion were associated with a 2.090 times higher risk of CVDs (OR = 2.090, 95% CI: 1.387–3.148). A history of heart disease in close relatives increased the risk of CVDs in COPD patients (OR = 2.944, 95% CI: 1.923–4.506). The risk of CVDs in COPD patients was increased in patients with higher levels of WBC (OR = 1.197, 95% CI: 1.082–1.324) and MONO (OR = 1.115, 95% CI: 1.034–1.204). Higher levels of PLT (OR = 0.996, 95% CI: 0.993–0.999) and LYM (OR = 0.826, 95% CI: 0.625–1.091) were associated with a reduced risk of CVDs in COPD patients (Table 4). The formula of the prediction model was: Logit (P) = Ln (P/1-P) = 0.208 male + 0.036 age +0.202 smoking+0.227 overweight + 0.368 blood transfusion+0.540 heart disease in close relatives + 0.180 WBC + 0.109 MONO - 0.004 PLT - 0.191 LYM.

**Figure 2:**
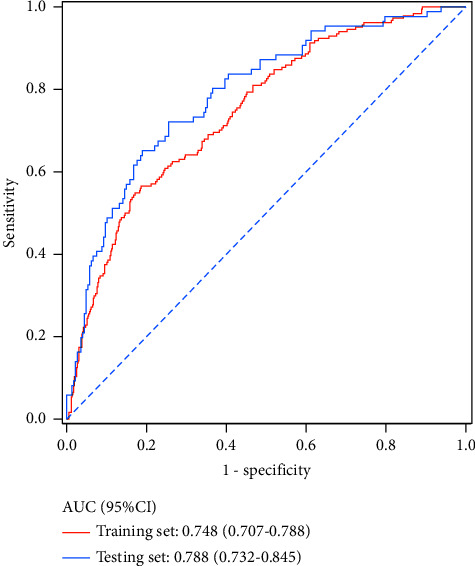
The ROC curves of predictive value of the training set and testing set.

**Figure 3:**
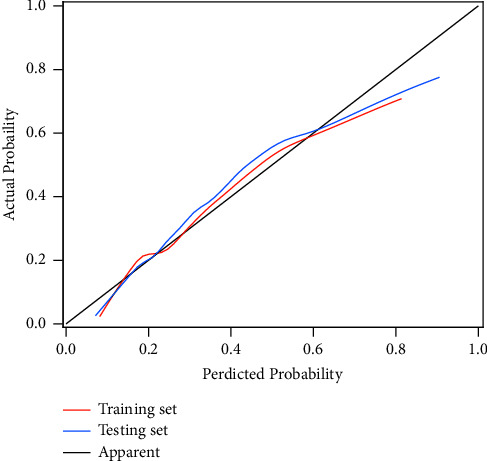
The calibration curves of the model in the training set and testing set.

**Figure 4:**
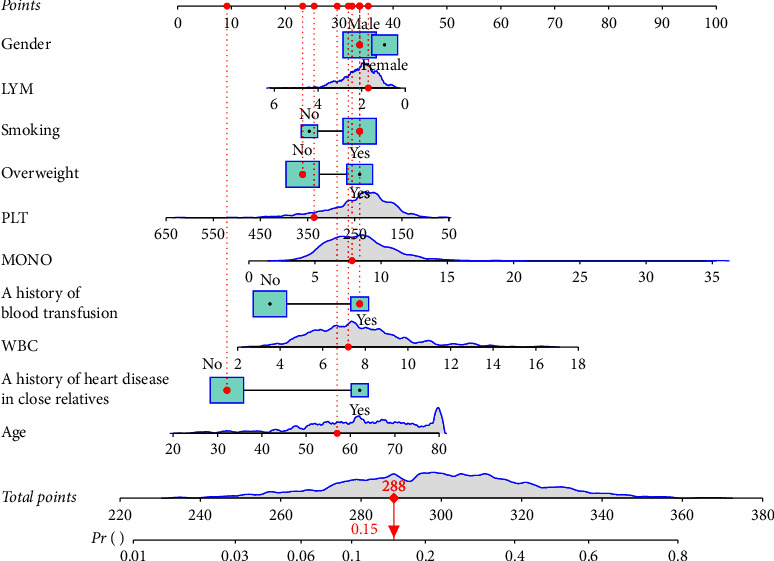
The nomogram for predicting the occurrence of CVDs in COPD patients.

The ROC curves of the training set and testing set are separately shown in Figure 2; the AUC value of the prediction model was 0.75 (95% CI: 0.71–0.79) in the training set and 0.79 (95% CI: 0.73–0.85) in the testing set. The accuracy was 0.76 (95% CI: 0.72–0.79) in the training set and 0.77 (95% CI: 0.72–0.82) in the testing set. The sensitivity was 0.56 (95% CI: 0.46–0.63) in the training set and 0.51 (95% CI: 0.41–0.62) in the testing set. The specificity was 0.83 (95% CI: 0.79–0.86) in the training set and 0.87 (95% CI: 0.83–0.92) in the testing set. The NPV was 0.84 (95% CI: 0.81–0.87) in the training set and 0.83 (95% CI: 0.78–0.87) in the testing set. The PPV was 0.53 (95% CI: 0.46–0.60) in the training set and 0.60 (95% CI: 0.49–0.71) in the testing set (Table 5). The calibration curves of the model in the training set and testing set are shown in Figure 3, which depict that the prediction values of the model in the training set and testing set deviated slightly from the perfected models, but were close to matching, indicating the prediction model had good agreement between the predictive probability and the actual probability. A nomogram was also established for predicting the occurrence of CVDs in COPD patients (Figure 4). A sample was randomly selected in the training set and the patient was a female without a history of heart disease in close relatives. The LYM level of the patient was 1.91 10^9^/L, the level of WBC was 7.12 10^9^/L, the level of PLT was 338 10^9^/L, and the level of MONO was 6.09 10^9^/L. The patient was 58 years old with a history of smoking and blood transfusion. The patient was not overweight. The total score was 288 and the possibility of CVDs in the patient was 0.15, which was similar with the actual results (Figure 4).

### 3.6. Subgroup Analysis of Prediction Ability of the Prediction Model

As there were two diagnosis methods for COPD patients in our study, subgroup analysis was performed in the testing set. The data delineated that the AUC value in COPD patients diagnosed by spirometry was 0.69 (95% CI: 0.53–0.85), the accuracy was 0.83 (95% CI: 0.75–0.88), the sensitivity was 0.38 (95% CI: 0.14–0.61), the specificity was 0.88 (95% CI: 0.82–0.93), the NPV was 0.92 (95% CI: 0.87–0.97), and the PPV was 0.27 (95% CI: 0.09–0.46). The AUC value in COPD diagnosed from the questionnaire was 0.72 (95% CI: 0.64–0.80), the accuracy was 0.63 (95% CI: 0.55–0.71), the sensitivity was 0.70 (95% CI: 0.60–0.81), the specificity was 0.61 (95% CI: 0.51–0.72), the NPV was 0.70 (95% CI: 0.60–0.81), and the PPV was 0.57 (95%CI: 0.45–0.68). Subgroup analysis was also performed based on whether the patients had a history of smoking. The AUC value in patients with a history of smoking was 0.72 (95% CI: 0.64–0.80), the accuracy was 0.63 (95% CI: 0.55–0.71), the sensitivity was 0.70 (95% CI: 0.60–0.81), the specificity was 0.61 (95% CI: 0.51–0.72), the NPV was 0.70 (95% CI: 0.60–0.81), the PPV was 0.57 (95% CI: 0.45–0.68). The AUC value in patients without a history of smoking was 0.64 (95% CI: 0.45–0.83), the accuracy was 0.72 (95% CI: 0.59–0.83), the sensitivity was 0.22 (95% CI: 0.01–0.49), the specificity was 0.82 (95% CI: 0.71–0.92), the NPV was 0.85 (95% CI: 0.75–0.95), and the PPV was 0.18 (95% CI: 0.01–0.41) (Table 6). The prediction model showed better performance in patients with COPD diagnosed according to questionnaires and patients with a history of smoking.

## 4. Discussion

This study collected the data of 1022 COPD patients with CVDs to evaluate the factors associated with the occurrence of CVDs in COPD patients and establish a prediction model based on these predictors. The data revealed that male, age, smoking history, overweight, history of blood transfusion or heart disease in close relatives, and levels of WBC, PLT, LYM, and MONO were predictors for CVDs in COPD patients. Additionally, we established a prediction model for the occurrence of CVDs in COPD patients based on these predictors, the AUC value of the prediction model was 0.75 in the training set and 0.77 in the testing set, which showed good predictive performance. Subgroup analysis revealed that the prediction model had better performance in patients with COPD diagnosed according to questionnaires and patients with a history of smoking.

Cigarette smoke is the major cause of COPD, which results in about 95% of COPD cases in industrialized countries [22]. Smoking is also reported to be one of the most important risk factors for COPD with CVDs [23]. This may be due to the diverse inflammatory responses resulted from smoking in COPD patients, which increased the risk of CVDs [24]. Austin et al. identified that alveolar macrophages in bronchoalveolar lavage from the lungs of smokers might release more reactive oxygen species than nonsmokers [25]. Herein, COPD patients with a history of smoking were associated with a higher risk of CVDs. Previously, several studies have indicated that age was associated with the incidence of CVDs in COPD patients [14, 26]. These findings supported the results in our study, which showed that increased age was correlated with a higher risk of CVDs in the COPD patients. In the current study, patients with a history of blood transfusion were also associated with an increased risk of CVDs in COPD patients. As reported, blood transfusion was a risk factor of major cardiovascular events in patients with acute myocardial infarction and anemia [27, 28]. Family history of a heart disease is widely proposed to be an essential marker for predicting the occurrence of cardiovascular events in patients [29, 30], which provide evidence to the findings of our study, which depicted that a history of heart disease in close relatives was associated with an increased risk of CVDs in COPD patients. Blood routine parameters are essential inflammatory markers of COPD and some of the inflammatory makers were also elevated in patients with COPD [31, 32]. MONO circulates in the blood, bone marrow, and spleen and are one of the active members of inflammation in COPD [33]. An increased WBC and a decreased LYM count were also identified in COPD patients compared to healthy subjects [34, 35]. Inflammation was associated with the changes in structure, shape, and dynamics of PLT, which may further affect atherogenic and thrombotic events [36]. Another study also showed that increased levels of inflammatory markers are associated with the increased incidence of atherosclerosis, coronary heart disease, congestive heart failure, and atrial fibrillation [37]. In our study, higher WBC and MONO levels were associated with an increased risk of CVDs in COPD patients while the higher levels of PLT and LYM were associated with a decreased risk of CVDs in COPD patients. For COPD patients, regular blood routine inspection should be conducted to pay close attention to the levels of WBC, MONO, PLT, and LYM for timely identifying patients with a high risk of CVDs.

The current study assessed the predictors for CVDs in COPD patients and established a prediction model based on these predictors in the training set. The validation of the prediction model was performed in the testing set. The AUC values of the model showed good predictive abilities in both the training set and the testing set. The calibration curves of the model also suggested that the prediction model had good agreement between the predictive probability and the actual probability, indicating our prediction model had good predictive performance. Previously, a prediction model for the occurrence of CVDs in COPD patients was constructed based on the MONO level/HDL cholesterol ratio, which showed an AUC value of 0.73, and our prediction model had better predictive performances than the model. Additionally, a nomogram was plotted in line with our model, which was easy for the clinicians to calculate the score directly from the graph and quickly estimate the possibility of CVDs in a COPD patient. The nomogram might offer a tool for the clinicians to provide timely interventions to prevent the occurrence of CVDs in patients with a high risk of CVDs.

The strengths of this study were that we dealt with the missing data and sensitivity analysis was conducted, which revealed that there was no significant difference between the characteristics of patients before and after manipulating the missing data. These suggested that the data used in our study through manipulating the missing data might reduce the bias than simply deleting the data, which might increase the reliability of our results. Internal validation was also performed to verify the results of the present study. Several limitations existed in the present study. First, the sample size was small and the statistical power was reduced. Second, external validation was not conducted. Third, some data collected from questionnaires in NHANES were self-reported, which might cause bias. In the future, studies with large scale of sample size were required to validate the findings of our study.

## 5. Conclusions

Herein, a prediction model was constructed for predicting CVDs in COPD patients based on predictors including gender, age, smoking history, overweight, history of blood transfusion or heart disease in close relatives, and levels of WBC, MONO, PLT, and LYM using the data of 1022 patients from NHANES database. The results showed that our prediction model had good predictive performance in predicting CVDs in COPD patients with AUC values of 0.75 in the training set and 0.79 in the testing set. A nomogram was also plotted for predicting the occurrence of CVDs in COPD patients. The findings might help identify COPD patients with a high risk of CVDs and provide timely interventions and treatment to prevent the occurrence of CVDs. For future work, we are planning to collect the samples of COPD in our hospital and use these data to verify the predictive performance of the prediction model. Meanwhile, advanced deep learning and optimization approaches will be employed to help improve the predictive value of predicting CVDs in COPD patients.

## Figures and Tables

**Table 1:** Comparison of the characteristics between the COPD patients with CVDs and without CVDs.

Variable	Total (*n* = 1022)	*CVD*		Statistical magnitude	*P*
		No (*n* = 752)	Yes (*n* = 270)		
*Gender, n (%)*				*χ * ^2^ = 0.012	0.912
Male	635 (62.13)	468 (62.23)	167 (61.85)		
Female	387 (37.87)	284 (37.77)	103 (38.15)		
Age, mean ± SD	61.92 ± 12.87	60.34 ± 13.20	66.33 ± 10.72	*t* = -7.38	<0.001
*Race, n (%)*				*χ * ^2^ = 1.323	0.516
Non-Hispanic White	699 (68.40)	507 (67.42)	192 (71.11)		
Non-Hispanic Black	170 (16.63)	130 (17.29)	40 (14.81)		
Other race	153 (14.97)	115 (15.29)	38 (14.07)		
*Education level, n (%)*				*Z* = -2.139	0.032
Under 12th grade	278 (27.20)	191 (25.40)	87 (32.22)		
High school grad/GED	274 (26.81)	203 (26.99)	71 (26.30)		
Some college or above	470 (45.99)	358 (47.61)	112 (41.48)		
*Marital status, n (%)*				*χ * ^2^ = 0.634	0.426
Married	536 (52.45)	400 (53.19)	136 (50.37)		
Others	486 (47.55)	352 (46.81)	134 (49.63)		
*Annual family income, n (%)*				*χ * ^2^ = 7.599	0.006
<20000$	318 (31.12)	216 (28.72)	102 (37.78)		
≥20000$	704 (68.88)	536 (71.28)	168 (62.22)		
*Smoking, n (%)*				*χ * ^2^ = 8.817	0.003
Yes	823 (80.53)	589 (78.32)	234 (86.67)		
No	199 (19.47)	163 (21.68)	36 (13.33)		
*Overweight, n (%)*				*χ * ^2^ = 25.203	<0.001
Yes	385 (37.67)	249 (33.11)	136 (50.37)		
No	637 (62.33)	503 (66.89)	134 (49.63)		
*A history of blood transfusion, n (%)*				*χ * ^2^ = 48.405	<0.001
Yes	237 (23.19)	133 (17.69)	104 (38.52)		
No	785 (76.81)	619 (82.31)	166 (61.48)		
*A history of heart disease in close relatives, n (%)*				*χ * ^2^ = 31.967	<0.001
Yes	211 (20.65)	123 (16.36)	88 (32.59)		
No	811 (79.35)	629 (83.64)	182 (67.41)		
WBC, mean ± SD	7.75 ± 2.25	7.62 ± 2.22	8.08 ± 2.30	*t* = -2.89	0.004
MONO, mean ± SD	8.31 ± 2.52	8.16 ± 2.52	8.75 ± 2.49	*t* = -3.30	<0.001
NEUT, *M* (*Q*_1_, *Q*_3_)	4.50 (4.10,5.60)	4.40 (4.00, 5.60)	4.80 (4.40, 6.00)	*Z* = 3.289	0.001
PLT, mean ± SD	240.39 ± 65.65	244.59 ± 65.97	228.70 ± 63.42	*t* = 3.43	<0.001
LYM, *M* (*Q*_1_, *Q*_3_)	2.00 (1.80, 2.50)	2.00 (1.90, 2.50)	1.90 (1.70, 2.40)	*Z* = -2.576	0.010
NLR, *M* (*Q*_1_, *Q*_3_)	2.26 (2.00, 3.11)	2.19 (1.97, 2.91)	2.55 (2.31, 3.57)	*Z* = 4.577	<0.001
PLR, *M* (*Q*_1_, *Q*_3_)	118.27 (109.05, 154.74)	119.18 (110.00, 155.19)	114.49 (106.50, 152.14)	*Z* = -0.371	0.711

COPD: chronic obstructive pulmonary disease, WBC: white blood cell, MONO: monocyte, NEUT: neutrophil, PLT: platelet, LYM: lymphocyte, NLR: neutrophil/lymphocyte ratio, PLR: platelet/lymphocyte ratio, CVD: cardiovascular disease, GED: general equivalent diploma or equivalent.

**Table 2:** Predictors of CVDs in COPD patients.

Variable	*β*	S.E	Wald	*P*	OR	Lower	Upper
Constant	−5.403	0.787	47.107	<0.001			
Gender (male)	0.059	0.175	0.112	0.738	1.060	0.752	1.494
Age	0.039	0.007	27.738	<0.001	1.040	1.025	1.055
Smoking (yes)	0.552	0.225	6.039	0.014	1.737	1.118	2.697
Overweight (yes)	0.687	0.161	18.172	<0.001	1.987	1.449	2.725
A history of blood transfusion (yes)	0.891	0.174	26.305	<0.001	2.437	1.734	3.425
A history of heart disease in close relatives (yes)	1.014	0.185	30.094	<0.001	2.758	1.919	3.962
WBC	0.205	0.044	21.317	<0.001	1.227	1.125	1.338
MONO	0.082	0.034	5.941	0.015	1.085	1.016	1.159
PLT	−0.004	0.001	10.282	0.001	0.996	0.993	0.998
LYM	−0.325	0.123	6.948	0.008	0.723	0.568	0.920

WBC: white blood cell, MONO: monocyte, PLT: platelet, LYM: lymphocyte, CVD: cardiovascular disease, COPD: chronic obstructive pulmonary disease.

**Table 3:** The equilibrium test of the training set and testing set.

Variable	Total (*n* = 1022)	Testing set (*n* = 313)	Training set (*n* = 709)	Statistical magnitude	*P*
*Gender, n (%)*				*χ * ^2^ = 0.120	0.729
Male	635 (62.13)	192 (61.34)	443 (62.48)		
Female	387 (37.87)	121 (38.66)	266 (37.52)		
Age, Mean ± SD	61.92 ± 12.87	61.56 ± 12.67	62.08 ± 12.96	*t* = −0.60	0.551
*Race, n (%)*				*χ * ^2^ = 0.812	0.666
Non-Hispanic White	699 (68.40)	220 (70.29)	479 (67.56)		
Non-Hispanic Black	170 (16.63)	50 (15.97)	120 (16.93)		
Other race	153 (14.97)	43 (13.74)	110 (15.51)		
*Education level, n (%)*				*Z* = −0.148	0.882
Under 12th grade	278 (27.20)	82 (26.20)	196 (27.64)		
High school grad/GED	274 (26.81)	91 (29.07)	183 (25.81)		
Some college or above	470 (45.99)	140 (44.73)	330 (46.54)		
*Marital status, n (%)*				*χ * ^2^ = 1.228	0.268
Married	536 (52.45)	156 (49.84)	380 (53.60)		
Others	486 (47.55)	157 (50.16)	329 (46.40)		
*Annual family income, n (%)*				*χ * ^2^ = 0.056	0.814
<20000$	318 (31.12)	99 (31.63)	219 (30.89)		
≥20000$	704 (68.88)	214 (68.37)	490 (69.11)		
*Smoking*, *n* (%)				*χ * ^2^ = 0.026	0.871
Yes	823 (80.53)	253 (80.83)	570 (80.39)		
No	199 (19.47)	60 (19.17)	139 (19.61)		
*Overweight, n (%)*				*χ * ^2^ = 0.023	0.879
Yes	385 (37.67)	119 (38.02)	266 (37.52)		
No	637 (62.33)	194 (61.98)	443 (62.48)		
*A history of blood transfusion, n (%)*				*χ * ^2^ = 0.758	0.384
Yes	237 (23.19)	78 (24.92)	159 (22.43)		
No	785 (76.81)	235 (75.08)	550 (77.57)		
*A history of heart disease in close relatives, n (%)*				*χ * ^2^ = 0.369	0.544
Yes	211 (20.65)	61 (19.49)	150 (21.16)		
No	811 (79.35)	252 (80.51)	559 (78.84)		
WBC, Mean ± SD	7.75 ± 2.25	7.77 ± 2.08	7.73 ± 2.32	*t* = 0.26	0.798
MONO, Mean ± SD	8.31 ± 2.52	8.19 ± 2.29	8.37 ± 2.62	*t* = −1.12	0.261
NEUT, *M* (*Q*_1_, *Q*_3_)	4.50(4.10,5.60)	4.60(4.22,5.70)	4.50(4.10,5.60)	*Z* = 1.110	0.267
PLT, Mean ± SD	240.39 ± 65.65	242.15 ± 62.85	239.62 ± 66.88	*t* = 0.57	0.569
LYM, *M* (*Q*_1_, *Q*_3_)	2.00 (1.80,2.50)	2.00 (1.80,2.40)	2.00 (1.80,2.50)	*Z* = −0.607	0.544
NLR, *M* (*Q*_1_, *Q*_3_)	2.26 (2.00,3.11)	2.34 (2.12,3.21)	2.24 (2.00,3.02)	*Z* = 1.246	0.213
PLR, *M* (*Q*_1_, *Q*_3_)	118.27 (109.05,154.74)	121.50 (112.27,157.50)	116.43 (107.69,152.50)	*Z* = 1.220	0.222
*CVD, n(%)*				*χ * ^2^ = 0.259	0.611
No	752 (73.58)	227 (72.52)	525 (74.05)		
Yes	270 (26.42)	86 (27.48)	184 (25.95)		

COPD: chronic obstructive pulmonary disease, WBC: white blood cell, MONO: monocyte, NEUT: neutrophil, PLT: platelet, LYM: lymphocyte, NLR: neutrophil/lymphocyte ratio, PLR: platelet/lymphocyte ratio, CVD: cardiovascular disease, GED: general equivalent diploma or equivalent.

**Table 4:** Construction of the logistic prediction model.

Variable	*β*	S.E	Wald	*P*	OR	Lower	Upper
Constant	−4.242	0.899	22.266	<0.001			
Gender (male)	0.208	0.209	0.987	0.321	1.231	0.817	1.856
Age	0.036	0.009	17.130	<0.001	1.037	1.019	1.054
Smoking (yes)	0.202	0.132	2.324	0.127	1.497	0.891	2.513
Overweight (yes)	0.227	0.096	5.557	0.018	1.575	1.080	2.298
A history of blood transfusion (yes)	0.368	0.105	12.418	<0.001	2.090	1.387	3.148
A history of heart disease in close relatives (yes)	0.540	0.109	24.725	<0.001	2.944	1.923	4.506
WBC	0.180	0.051	12.230	<0.001	1.197	1.082	1.324
MONO	0.109	0.039	7.903	0.005	1.115	1.034	1.204
PLT	−0.004	0.002	6.603	0.010	0.996	0.993	0.999
LYM	−0.191	0.142	1.817	0.178	0.826	0.625	1.091

WBC: white blood cell, MONO: monocyte, PLT: platelet, LYM: lymphocyte.

**Table 5:** The predictive performance of the model.

Characteristic	Training set (95%CI)	Testing set (95%CI)
AUC	0.75 (0.71–0.79)	0.79 (0.73–0.85)
Accuracy	0.76 (0.72–0.79)	0.77 (0.72–0.82)
Sensitivity	0.56 (0.46–0.63)	0.51 (0.41–0.62)
Specificity	0.83 (0.79–0.86)	0.87 (0.83–0.92)
NPV	0.84 (0.81–0.87)	0.83 (0.78–0.87)
PPV	0.53 (0.46–0.60)	0.60 (0.49–0.71)

AUC: the area under the curve, NPV: negative predictive value, PPV: positive predictive value.

**Table 6:** Subgroup analysis of the predictive ability of the prediction model.

Subgroup	Characteristic	Testing set (95%CI)
*COPD diagnosed by spirometry*		
	AUC	0.69 (0.53–0.85)
	Accuracy	0.83 (0.75–0.88)
	Sensitivity	0.38 (0.14–0.61)
	Specificity	0.88 (0.82–0.93)
	NPV	0.92 (0.87–0.97)
	PPV	0.27 (0.09–0.46)

*COPD diagnosed from the questionnaire*		
	AUC	0.72 (0.64–0.80)
	Accuracy	0.63 (0.55–0.71)
	Sensitivity	0.70 (0.60–0.81)
	Specificity	0.61 (0.51–0.72)
	NPV	0.70 (0.60–0.81)
	PPV	0.57 (0.45–0.68)

*Smoking (yes)*		
	AUC	0.79 (0.73–0.85)
	Accuracy	0.73 (0.67–0.79)
	Sensitivity	0.65 (0.54–0.76)
	Specificity	0.76 (0.70–0.83)
	NPV	0.84 (0.78–0.90)
	PPV	0.54 (0.44–0.64)

*Smoking (no)*		
	AUC	0.64 (0.45–0.83)
	Accuracy	0.72 (0.59–0.83)
	Sensitivity	0.22 (0.01–0.49)
	Specificity	0.82 (0.71–0.92)
	NPV	0.85 (0.75–0.95)
	PPV	0.18 (0.01–0.41)

AUC: the area under the curve, NPV: negative predictive value, PPV: positive predictive value.

## Data Availability

The datasets generated and/or analyzed during the current study are available via https://www.cdc.gov/nchs/nhanes/index.htm.

## Supplementary Materials

Supplementary Materials Supplementary Table 1 Sensitivity analysis of the missing data before or after manipulation.
