# Supplementary material for: A Nomogram for Predicting Cardiovascular Diseases in Chronic Obstructive Pulmonary Disease Patients
Source: J Healthc Eng. 2022 Oct 18;2022:6394290. doi: 10.1155/2022/6394290 (PMC9596246; doi:10.1155/2022/6394290)
Supplement: Supplementary Materials — Supplementary Table 1 Sensitivity analysis of the missing data before or after manipulation. [file 6394290.f1.docx]

Supplementary Table 1 Sensitivity analysis of the missing data before or after manipulation

| Variable | n (%) | After filling (n=1022) | Before filling (n=1022) | Statistical magnitude | *P* |
| --- | --- | --- | --- | --- | --- |
| Gender | 0 (0.00) |  |  |  |  |
| Age | 0 (0.00) |  |  |  |  |
| Race | 0 (0.00) |  |  |  |  |
| Education | 0 (0.00) |  |  |  |  |
| Marital status | 0 (0.00) |  |  |  |  |
| Annual family income, n (%) | 18 (1.76) |  |  | χ^2^=0.014 | 0.907 |
| <20000$ |  | 318 (31.12) | 310 (30.88) |  |  |
| ≥20000$ |  | 704 (68.88) | 694 (69.12) |  |  |
| Smoking | 0 (0.00) |  |  |  |  |
| Overweight | 0 (0.00) |  |  |  |  |
| A history of blood transfusion | 0 (0.00) |  |  |  |  |
| A history of heart disease in close relatives | 0 (0.00) |  |  |  |  |
| WBC, Mean ± SD | 63 (6.16) | 7.75 ± 2.25 | 7.75 ±2 .31 | t=-0.01 | 0.993 |
| MONO, Mean ± SD | 64 (6.26) | 8.31 ± 2.52 | 8.30 ± 2.58 | t=0.10 | 0.924 |
| NEUT, M (Q_1_, Q_3_) | 64 (6.26) | 4.50(4.10, 5.60) | 4.50 (4.10, 5.70) | Z=-0.214 | 0.830 |
| PLT, Mean ± SD | 63 (6.16) | 240.39 ± 65.65 | 241.27 ± 67.23 | t=-0.29 | 0.768 |
| LYM, M (Q_1_, Q_3_) | 64 (6.26) | 2.00 (1.80, 2.50) | 2.00 (1.80, 2.50) | Z=-0.212 | 0.832 |
| NLR, M (Q_1_, Q_3_) | 64 (6.26) | 2.26 (2.00, 3.11) | 2.25 (2.00, 3.13) | Z=-0.412 | 0.681 |
| PLR, M (Q_1_, Q_3_) | 64 (6.26) | 118.27 (109.05, 154.74) | 118.46 (109.00, 156.11) | Z=-0.048 | 0.962 |
| CVD | 0 (0.00) |  |  |  |  |

WBC: white blood cell, MONO: monocyte, NEUT: neutrophil, PLT: platelet, LYM: lymphocyte, NLR: neutrophil /lymphocyte ratio, PLR: platelet/lymphocyte ratio, CVD: cardiovascular disease
